# Oleate Prevents Palmitate-Induced Atrophy via Modulation of Mitochondrial ROS Production in Skeletal Myotubes

**DOI:** 10.1155/2017/2739721

**Published:** 2017-08-30

**Authors:** Hojun Lee, Jae-Young Lim, Seung-Jun Choi

**Affiliations:** ^1^Division of Sports and Health Science, Kyungsung University, Busan, Republic of Korea; ^2^Mechanical & Molecular Myology Lab, Department of Rehabilitation Medicine, College of Medicine, Seoul National University Bundang Hospital, Seongnam, Republic of Korea

## Abstract

Accumulation of saturated fatty acids contributes to lipotoxicity-related insulin resistance and atrophy in skeletal muscle. Conversely, unsaturated fatty acids like docosahexaenoic acid were proven to preserve muscle mass. However, it is not known if the most common unsaturated oleate will protect skeletal myotubes against palmitate-mediated atrophy, and its specific mechanism remains to be elucidated. Therefore, we investigated the effects of oleate on atrophy-related factors in palmitate-conditioned myotubes. Exposure of myotubes to palmitate, but not to oleate, led to an induction of fragmented nuclei, myotube loss, atrophy, and mitochondrial superoxide in a dose-dependent manner. Treatment of oleate to myotubes attenuated production of palmitate-induced mitochondrial superoxide in a dose-dependent manner. The treatment of oleate or MitoTEMPO to palmitate-conditioned myotubes led to inhibition of palmitate-induced mRNA expression of proinflammatory (TNF-*α* and IL6), mitochondrial fission (Drp1 and Fis1), and atrophy markers (myostatin and atrogin1). In accordance with the gene expression data, our immunocytochemistry experiment demonstrated that oleate and MitoTEMPO prevented or attenuated palmitate-mediated myotube shrinkage. These results provide a mechanism indicating that oleate prevents palmitate-mediated atrophy via at least partial modulation of mitochondrial superoxide production.

## 1. Introduction

The loss of skeletal muscle mass and integrity is associated with the development of a variety of diseases, including type 2 diabetes, obesity, and sarcopenia [1–4]. Skeletal muscle is a highly organized tissue with tremendous plasticity, responding and adapting to nutritional challenges by regulating its mass and metabolic properties [5–7]. The process of skeletal muscle synthesis and degradation is modulated by various lipid-related factors [8, 9]. Since skeletal muscle is embedded with abundant mitochondria utilizing lipid as a substrate greatly, the roles of excess lipids are under active research in the field of muscle physiology. An increased level of plasma-free fatty acids is linked with insulin resistance in skeletal muscle [10, 11]. The exposure of differentiated skeletal myotubes to saturated palmitic acid leads to lipotoxicity-mediated myofiber loss [12]. Specifically, reactive oxygen species (ROS) from mitochondria overburdened with lipids induces apoptosis [13, 14]. Due to the nature of high levels of skeletal muscle locomotion, mitochondria within myofibers require a constant supply of oxygen to produce ATP via fatty acid-mediated oxidative phosphorylation, which confers myofibers susceptible to mitochondrial ROS [15].

Several lines of evidence proved that the chemical structure of intracellular fatty acid is an important factor if it is detrimental to skeletal muscle function. Palmitate, the most common saturated fatty acid, is coupled with the expression of inflammatory cytokines leading to insulin-resistant states [16–20], while oleate, the most abundant unsaturated fatty acid, improves metabolic properties via deacetylation of PGC1*α*, a master regulator of mitochondrial biogenesis [21, 22], suggesting a possibility of the protective role of oleate in palmitate-induced muscle atrophy. Although a study demonstrated that an omega-3 unsaturated fatty acid, docosahexaenoic acid (DHA), preserves skeletal myotube integrity [23], the mechanisms by which unsaturated fatty acid protects myotube against palmitate-induced atrophy remain elusive. Moreover, since a previous relevant study was tested in a condition of high dose of palmitate, which induced significant loss of myotubes during the experiment [23], an optimal concentration of palmitate for induction of atrophy without loss of myotubes is warranted to unravel saturated fatty acid-mediated atrophy mechanisms.

In skeletal muscle, increased flux of fatty acids contributes to the production of mitochondrial ROS [24]. Multiple studies suggested evidences that overproduced mitochondrial superoxide from electron transport chain is a significant initiator in the induction of canonical Nf-*κ*B pathway-mediated TNF-*α* production, coupled with a variety of muscle-related pathogenesis [25, 26]. In line with this, oxidative stress induces mitochondrial dysfunction in skeletal muscle in a vicious and positive feedback loop manner [27, 28]. Based on a previous study that the mitochondrial redox state is tightly coupled with skeletal muscle-related pathogenesis [29], it is hypothesized that fatty acid-mediated modulation of mitochondrial ROS is a critical factor in the regulation of skeletal muscle size. Although the protective effect of oleate against mitochondrial dysfunction and inflammation was identified in palmitate-conditioned neuronal cells [30], it remains still elusive as to whether oleate would protect myotubes against palmitate-induced atrophy. Therefore, the main purpose of this study is twofold: to investigate if oleate would attenuate or prevent palmitate-mediated muscle atrophy and if oleate mediated-inhibition of mitochondrial ROS would reduce palmitate-induced atrophy via modulation of mitochondrial fission and proinflammatory cytokines.

## 2. Materials and Methods

### 2.1. Free Fatty Acid Preparation

Palmitate-containing medium was prepared by incubation of palmitate with DMEM supplemented with 2% FFA-free BSA as described previously with a minor modification [31].

Briefly, sodium palmitate (Sigma-Aldrich) was dissolved to make a 500 mM stock solution in 50% ethanol and was incubated in a water bath at 50°C for 1 hour. The tubes containing sodium palmitate were inverted every 10 minutes for complete dissolution. Before application to the myotubes, palmitate was conjugated to bovine serum albumin (BSA) at 37°C by diluting with differentiation medium containing 2% fat-free BSA (Bovogen). Premade oleic acid solution free of ethanol was purchased from Sigma-Aldrich. All experiments were performed in medium containing 0.1% ethanol.

### 2.2. Cell Culture and Treatment Condition

C2C12 myoblasts (ATCC) were seeded onto collagen-coated 6-well plates and were maintained in Dulbecco's modified Eagle's medium (DMEM) containing 10% fetal bovine serum (Welgene, Korea), 100 units/mL penicillin, and 100 mg/mL streptomycin (Welgene, Korea) in a humidified atmosphere of 95% air and 5% CO2 at 37°C. When myoblasts were confluent (95%), growth medium was changed to differentiation medium (DM) supplemented with 2% horse serum, 100 units/mL penicillin, and 100 mg/mL streptomycin (Welgene, Korea) and was incubated for 96 hours for myogenic differentiation. As indicated in each figure, dose- and time-dependent responses of palmitate and oleate were tested from 0.1 to 1 mM, respectively. In subsequent experiments, 0.3 mM palmitate or a mixture of 0.3 mM palmitate and 0.5 mM oleate was incubated for 30 hours to test the preventive effect of oleate. A subset of the palmitate-conditioned myotubes was treated with 25 nM of MitoTEMPO (Sigma-Aldrich) to scavenge mitochondrial superoxide 1 h prior to palmitate treatment.

### 2.3. Immunocytochemistry

Myotubes were fixed and permeabilized by incubating in ice-cold methanol for 10 minutes. After rehydration in DPBS three times, the myotubes were incubated in blocking solution for 30 minutes at room temperature, followed by incubation in 2% BSA solution containing anti-sarcomeric myosin antibody MF-20 conjugated with Alexa Fluor 488 (eBioscience). After myonuclei were stained with DAPI (Molecular Probes), micrographs were acquired under a fluorescence microscope (Axioimager, Zeiss). The cells with a minimum number of three myonuclei were counted as differentiated myotubes.

### 2.4. MitoSOX Staining

Mitochondrial superoxide was stained with MitoSOX (Molecular Probes). The staining was performed according to the manufacture's protocol. Briefly, at the indicated time points, live myotubes were washed with DPBS twice and were incubated with 500 nM MitoSOX red for 30 minutes at 37°C. After washing with DPBS twice, the stained myotubes were maintained with MEM without Phenol red during the experimental period. The fluorescence intensity was analyzed and quantified using the ImageJ software (NIH).

### 2.5. MitoTracker Green Staining

Mitochondria were stained with MitoTracker Green (Molecular Probes). The staining was performed according to the manufacturer's protocol with minor modification. Briefly, at the indicated time points, live myotubes were washed with DPBS and were incubated with 200 nM of MitoTracker Green for 30 minutes in prewarmed buffer in a 37°C cell culture incubator. The fluorescence intensity was analyzed and quantified using the ImageJ software (NIH).

### 2.6. qRT-PCR

Differentiated myotubes were washed with cold DPBS and lysed with TRIzol reagent. Chloroform was added for the separation of RNA from DNA and protein fractions. RNA fraction was precipitated by the addition of isopropanol, followed by centrifugation at 12,000*g* for 8 minutes at 4°C. The RNA pellet was washed with 75% ethanol and centrifuged at 12,000*g* for 5 minutes at 4°C. After removal of ethanol, RNA pellets were air dried before resuspending with RNAse-free water. The RNA concentration was quantified using a NanoDrop spectrophotometer. Reverse transcription was performed with a cDNA synthesis kit according to manufacturer's instruction (Bioneer, Korea). Following cDNA synthesis, RT-qPCR was performed in a Rea-Time PCR system (Applied Biosystems) using a SYBR Green master mix (Bioline, Korea) and primer pair sets described in Table 1. Cycle threshold (Ct) values were normalized to the housekeeping gene (HPRT1-F: 5′- GACTTGCTCGAGATGTCATG -3′, HPRT1-R: 5′- TACAGTCATAGGAATGGACC -3′).

### 2.7. Statistics

The results were presented as mean ± SE for a minimum of three independent experiments. One-way or two-way ANOVA was followed by Tukey's post hoc test to present statistical difference among groups. The statistical significance was set at *P* < 0.05.

## 3. Results

### 3.1. The Effects of Palmitate and Oleate on Myotube Morphology

**Figure 1:**
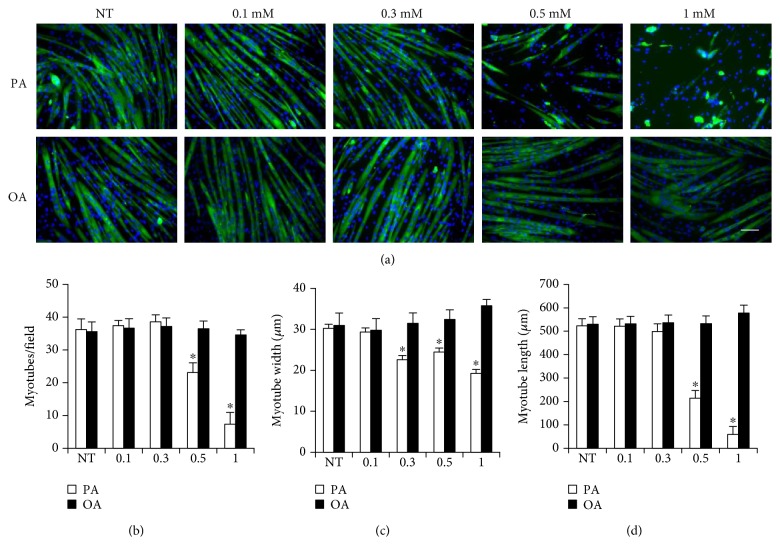
The effects of palmitic and oleic acid on morphological features of myotubes. Differentiated C2C12 myotubes were treated with 0.1, 0.3, 0.5, and 1 mM palmitate or oleate for 36 h. (a) Differentiated myotubes were stained with MF20 antibody and were visualized by florescence microscope (magnification = ×20). (b) Myotube number, (c) width, and (d) length were quantified. Bar graph represented means ± SE. Scale bar = 100 *μ*m. The data were analyzed using two-way ANOVA followed by Tukey's post hoc test. ^∗^*p* < 0.05 versus NT. NT: nontreatment.

Dose-response experiments were conducted to determine the effects of different doses of palmitate and oleate on differentiated myotube morphology (Figure 1). Immunocytochemistry experiments demonstrated that treatment of palmitate led to a decrease in number, width, and length of myotubes in a dose-dependent manner while oleate has no significant effect on myotube morphology in a 30-hour incubation. Especially, the treatment of 0.3 mM palmitate led to reduction of width and length in myotubes in the absence of significant myotube loss.

### 3.2. The Effects of Palmitate and Oleate on Myonucleus Number and Fragmentation

**Figure 2:**
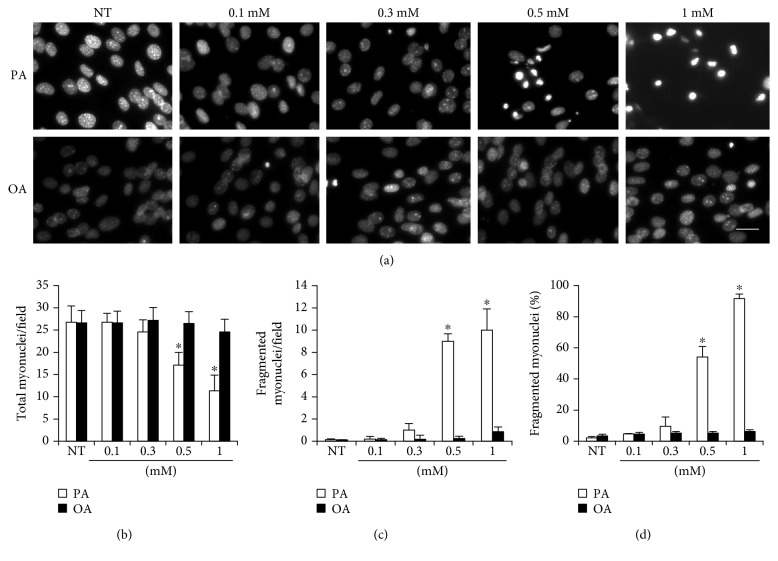
The effects of palmitic and oleic acid on morphological features of myonuclei. Differentiated C2C12 myotubes were treated with 0.1, 0.3, 0.5, and 1 mM palmitate or oleate for 36 h. (a) Myonuclei were stained with DAPI and were visualized by florescence microscope (magnification = ×64). (b) Total myonucleus number, (b) fragmented myonuclei, and (c) fragmented myonuclei (%) were quantified. Bar graph represented means ± SE. Scale bar = 20 *μ*m. The data were analyzed using two-way ANOVA followed by Tukey's post hoc test. ^∗^*p* < 0.05 versus NT. NT: nontreatment.

Myonuclei in the process of condensation are considered as a significant apoptotic sign of cells. The treatment of palmitate induced an increase in fragmented myonuclei in a dose-dependent manner when the concentration exceeded 0.3 mM (Figure 2). Therefore, a standard palmitate concentration was determined at 0.3 mM for further experiments to induce myotube atrophy in the absence of significant apoptosis. Oleate had no significant effects on myonucleus number and fragmentation.

### 3.3. The Effects of Oleate on Palmitate-Induced Mitochondrial Superoxide

**Figure 3:**
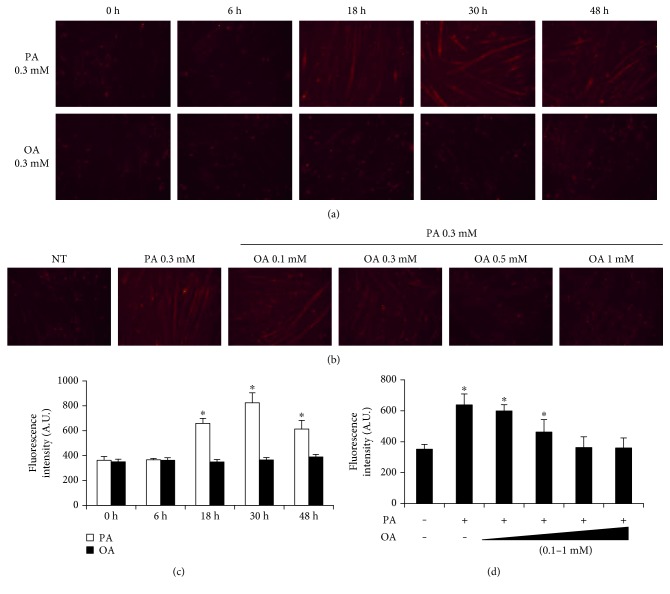
The effects of palmitic and oleic acid on mitochondrial superoxide production. (a) Differentiated C2C12 myotubes were treated with 0.3 mM palmitate or 0.3 mM oleate at the indicated time points. Differentiated myotubes were stained with MitoSOX. (b) Differentiated myotubes were cotreated with 0.3 mM palmitate and different doses of oleate (0.1, 0.3, 0.5, and 1 mM) for 30 h and were visualized by florescence microscope (magnification = ×20). (c) Dose effects of palmitic and oleic acid and (d) protective effects of oleic acid in palmitic acid-treated myotubes were quantified. Bar graph represented means ± SE. Scale bar = 100 *μ*m. The data were analyzed using one or two-way ANOVA followed by Tukey's post hoc test. ^∗^*p* < 0.05 versus NT. NT: non-treatment.

Mitochondrial superoxide was stained with MitoSOX in live myotubes and observed under florescence microscopy (Figure 3). Myotubes were treated with 0.3 mM palmitate and oleate. When incubated in 0.3 mM palmitate, the staining intensity was increased on an 18-hour incubation. The increased staining intensity persisted at the end of experiment. On the contrary, the staining intensity was not changed in myotubes treated with 0.3 mM oleate. To identify if the treatment of oleate was sufficient to mitigate palmitate-induced mitochondrial ROS, different doses of oleate were applied to myotubes conditioned in 0.3 mM palmitate for 30 hours. The addition of oleate to the palmitate treatment attenuated MitoSOX staining in a dose-dependent manner. The protective effect of oleate was pronounced when the concentration exceeded 0.5 mM. Therefore, 0.5 mM oleate was used to test the protective effect of oleate against palmitate in subsequent experiments.

### 3.4. The Effects of MitoTEMPO and Oleate on Mitochondrial Mass and Gene Expression of Mitochondrial Biogenesis and Dynamics in Palmitate-Treated Myotubes

**Figure 4:**
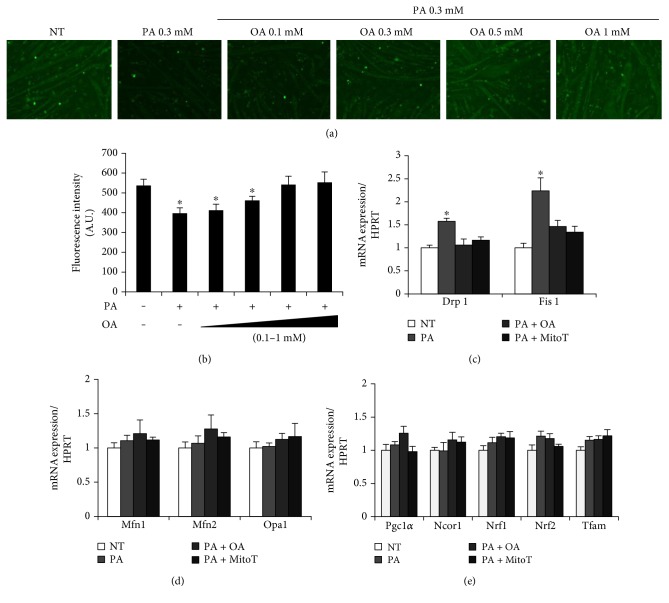
The effects of oleic acid and MitoTEMPO on mitochondrial biogenesis and dynamics in PA-treated myotubes. (a) Differentiated myotubes were cotreated with 0.3 mM palmitate and different doses of oleate (0.1, 0.3, 0.5, and 1 mM) for 30 h. The myotubes were visualized by florescence microscope after MitoTracker staining. (b) Florescence intensity of MitoTracker was quantified. (c) Gene expressions of mitochondrial fission, (d) fusion, and (e) biogenesis were quantified. Differentiated myotubes were cotreated with 0.3 mM palmitate and/or 0.5 mM oleate for 30 h. For some cells, 25 nM of MitoTEMPO was treated 1 h prior to palmitate treatment. Bar graph represented means ± SE. Scale bar = 100 *μ*m. The data were analyzed using one-way ANOVA followed by a Tukey's post hoc test. ^∗^*p* < 0.05 versus NT. NT: nontreatment; PA: palmitic acid; PA + OA: palmitic acid with oleic acid; PA + MitoT: palmitic acid + MitoTEMPO.

Mitochondrial biogenesis and dynamics are known to be an important regulator in skeletal muscle remodeling and atrophy. To determine the possible protective role of oleate in palmitate-treated myotubes, 0.3 mM palmitate along with different doses of oleate was applied to myotubes. Palmitate treatment decreased mitochondrial mass compared to the NT group, as evidenced by MitoTracker Green. The palmitate-mediated mitochondrial mass reduction was attenuated by cotreatment with oleate in a dose-dependent manner (Figures 4(a) and 4(b)). To determine if reduced mitochondria mass was related with the expression of mitochondrial biogenesis, fusion, and fission, relevant genes were tested using qRT-PCR. Palmitate increased the expression of mitochondrial fission markers (Drp-1 and Fis1) while the cotreatment with mitochondrial antioxidant MitoTEMPO or oleate attenuated all of these to the NT group (Figure 4(c)). The gene expressions of mitochondrial biogenesis (Pgc1-*α*, Ncor1, Nrf1, Nrf2, and Tfam) and fusion (Mfn1, Mfn2, and Opa1) were unaltered in all groups tested (Figures 4(d) and 4(e)). These results implicate a possibility that a palmitate-mediated reduction in mitochondrial mass originates from an increase in mitochondrial fission, not a decrease in mitochondrial biogenesis.

### 3.5. The Effects of MitoTEMPO and Oleate on Proinflammatory Cytokines in Palmitate-Treated Myotubes

**Figure 5:**
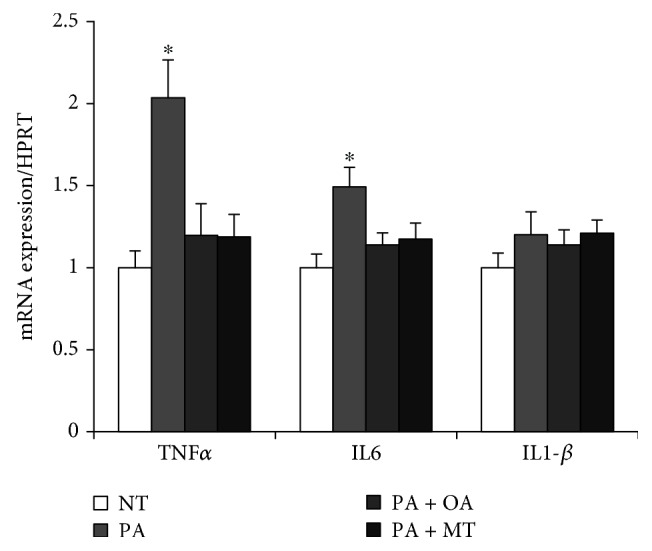
The effects of oleic acid and MitoTEMPO on the expression of proinflammatory cytokines in PA-treated myotubes. Differentiated myotubes were cotreated with 0.3 mM palmitate and/or 0.5 mM oleate for 30 h. For some cells, 25 nM of MitoTEMPO was treated 1 h prior to palmitate treatment. The gene expressions of TNF-*α*, IL6, and IL1-*β* were quantified. Bar graph represented means ± SE. The data were analyzed using one-way ANOVA followed by Tukey's post hoc test. ^∗^*p* < 0.05 versus NT. NT: nontreatment; PA: palmitic acid; PA + OA: palmitic acid with oleic acid; PA + MitoT: palmitic acid with MitoTEMPO.

Since several proinflammatory cytokines are tightly related with mitochondrial fission, the expression of TNF-*α*, IL6, and IL-*β* was quantified. Palmitate increased the expression of TNF-*α* and IL6 while the cotreatment with mitochondrial antioxidant MitoTEMPO or oleate attenuated all of these to the NT group. The gene expression of IL-*β* was unaltered in all groups tested (Figure 5).

### 3.6. The Effects of MitoTEMPO and Oleate on Morphological Changes and the Expression of Atrogenes in Palmitate-Treated Myotubes

**Figure 6:**
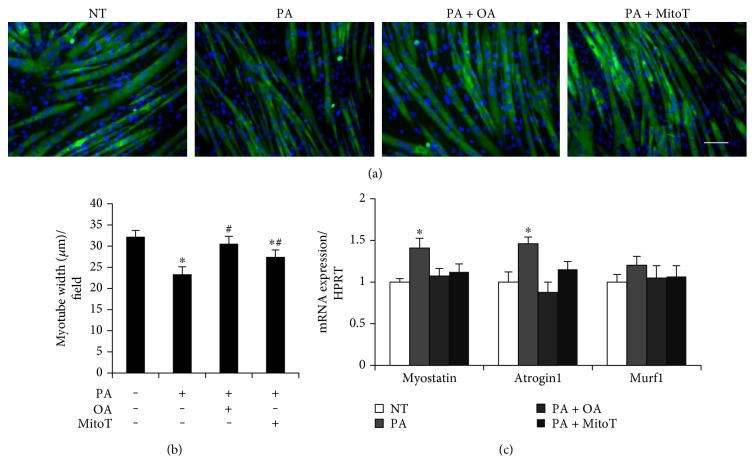
The effects of oleic acid and MitoTEMPO on morphological features of myotubes treated with PA. (a) Differentiated myotubes were cotreated with 0.3 mM palmitate and/or 0.5 mM oleate for 30 h. For some cells, 25 nM of MitoTEMPO was treated 1 h prior to palmitate treatment. Differentiated myotubes were stained with MF20 antibody and were visualized by florescence microscope (magnification = ×20). (b) Myotube width was quantified. (c) The expression of atrogenes was quantified. Bar graph represented means ± SE. Scale bar = 100 *μ*m. The data were analyzed using one-way ANOVA followed by Tukey's post hoc test. ^∗^*p* < 0.05 versus NT, ^#^*p* < 0.05 versus PA. NT: nontreatment; PA: palmitic acid; PA + OA: palmitic acid with oleic acid; PA + MitoT: palmitic acid with MitoTEMPO.

Immunocytochemistry was performed to investigate myotube morphology. Exposure to palmitate induces a reduction in myotube width, which is attenuated by the coincubation with MitoTEMPO or oleate (Figures 6(a) and 6(b)). And myotube length was not different among all groups tested (data not shown). The exposure of myotubes to palmitate induced the expression of myostatin and atrogin1 while the cotreatment with MitoTEMPO or oleate attenuated these to the NT group. Murf1 expression was unaltered in all groups tested (Figure 6(c)).

## 4. Discussion

The maintenance of skeletal muscle integrity is important for proper functioning of the musculoskeletal system, which is significantly affected by the nutritional status [8, 32]. Recent studies showed that various fatty acids are significantly linked with skeletal muscle functional properties [33, 34]. In the study, two most abundant fatty acids, palmitate and oleate, were investigated to unravel a mechanism by which these two fatty acids affect skeletal myotube atrophy. The major findings of this study were that oleate was protective against the negative effects of palmitate on skeletal myotube atrophy and this phenomenon was mediated via modulation of mitochondrial ROS, as further evidenced by mitochondrial fission, proinflammatory cytokines, and atrogenes.

In contrast to no observed effect of oleate, a high concentration of palmitate treatment (>0.5 mM) induced significant myotube loss and myonucleus fragmentation in a dose-dependent manner (Figures 1 and 2), supporting previous studies that saturated fatty acid, palmitate, is detrimental to skeletal muscle integrity [12, 21] while oleate has no effect on myotube morphology [35]. Since the treatment of 0.3 mM palmitate to myotubes for 30 hours led to an induction of sufficient atrophy in the absence of significant myonucleus fragmentation (Figures 1 and 2), 0.3 mM palmitate was used in subsequent experiments to avoid significant myotube loss, which is considered necessary for *in vitro* atrophy study with a minimal effect on the viability of the myotubes.

To determine if the fatty acid-mediated changes in myotube morphology were coupled with mitochondrial ROS production, the level of mitochondrial ROS was measured by MitoSOX staining in both oleate- and palmitate-conditioned myotubes (Figures 3(a) and 3(b)). In line with our previous results, mitochondrial ROS production was increased following palmitate incubation in a time-dependent manner while there was no effect of oleate on the mitochondrial redox state. Interestingly, the coincubation of oleate attenuated palmitate-induced mitochondrial superoxide production in a dose-dependent manner. This suggests that oleate modulates the mitochondrial redox system, which is further evidenced by a previous study showing that oleate conditioning prevents palmitate-induced mitochondrial superoxide production in neuronal cells [30].

Mitochondrion is a dynamic subcellular organelle modulating its size and mass via biogenesis, fusion, and fission as necessary [36, 37]. Since reduced mitochondrial functional properties are coupled with skeletal and diaphragmatic muscle atrophy [38], the markers of mitochondrial mass, biogenesis, and dynamics were measured. Palmitate decreased mitochondrial mass while the cotreatment with oleate or MitoTEMPO inhibited the negative effect of palmitate (Figures 4(a) and 4(b)). In line with this, palmitate led to an induction of mitochondrial fission expressions (Drp-1, Fis-1) while the coincubation with oleate or MitoTEMPO normalized these to the basal level (Figure 4(c)). Mitochondria are accounted for more than 20% volume density in myofibers, which play a critical role in muscle's functional properties [39–41]. In response to overburden metabolic environment, mitochondria undergo morphology transitions modulated by dynamic processes of membrane fission, fusion, and biogenesis [42, 43]. These events of dynamics mediated by mitochondrial ROS are considered to be central regulators of the whole cellular activity [44]. Specifically, increased mitochondrial ROS is a critical inducer to mitochondrial fission in skeletal muscle [45], which is linked with atrophy [46]. Furthermore, the inhibition of Drp1-mediated mitochondrial fission prevents the loss of the mitochondrial membrane potential, inducing a cell survival mechanism [47]. Since the preventive effect of oleate on the expression of Drp-1 and Fis1 was observed in palmitate-conditioned myotubes, it is suggested that oleate inhibited mitochondrial fission through the prevention of mitochondrial ROS overproduction. In the study, the gene expressions of mitochondrial biogenesis (Pgc1-*α*, Ncor1, Nrf1, Nrf2, and Tfam) and fusion (Mfn1, Mfn2, and Opa1) were unaltered by palmitate treatment (Figure 4(e)). These results suggest that the reduced mass of mitochondria was mainly induced by increased mitochondrial fission, but not by reduction of their biogenesis under our experimental condition. It was originally hypothesized that the treatment of palmitate would decrease mitochondrial biogenesis-related factors because a previous study proved negative effects of a high concentration of palmitate (0.75 mM) on mitochondrial function in skeletal muscle [21]. On the contrary, a study also suggested that a low concentration of palmitate (0.1 mM) increased mitochondrial functional properties [48]. These discrepant results suggest that palmitate might possess a concentration-dependent biphasic effect on mitochondrial functional properties, which warrants a future study to unveil a mechanism to link palmitate the concentration-dependent mitochondrial state to skeletal muscle atrophy.

It is documented that proinflammatory activation is a prelude to mitochondrial fission and skeletal muscle atrophy [49–51]. Thus, the experiment was conducted to identify if the gene expression patterns of proinflammatory cytokines coincided with our mitochondrial fission data. Palmitate increased the expressions of TNF-*α* and IL6 while the cotreatment with oleate or MitoTEMPO prevented the effect of palmitate (Figure 5), which indeed suggests that oleate has an anti-inflammatory effect in palmitate-conditioned myotubes [52]. Contrary to our hypothesis, the expression level of IL1-*β* was unchanged by the palmitate treatment. Based on previous studies showing that 0.75 mM palmitate induces IL1-*β*-mediated apoptosis in mouse skeletal muscle [53, 54], it is conceivable that IL1-*β* might be involved in severe atrophy condition, but not in a condition causing mild atrophy without affecting the viability of myotubes.

Lastly, our data led us to investigate if the measured gene expression patterns would correspond to atrophy factors. Although Murf1 did not reach statistical significance, the expression levels of myostatin and atrogin1, known atrogenes, were increased in palmitate-conditioned myotubes, which were prevented by the coincubation of oleate or MitoTEMPO (Figure 6(c)). In line with this, our immunocytochemistry results revealed the complete prevention of palmitate-mediated myotube atrophy under the treatment of oleate. Although significant attenuation of myotube atrophy was observed, the treatment of MitoTEMPO proved not as effective as oleate (Figures 6(a) and 6(b)), which raises a possibility that mitochondrial ROS might not be a sole contributor to oleate-mediated atrophy prevention. With regard to muscle physiology, even potent proinflammatory molecule such as TNF-*α* alone is not sufficient to induce protein loss [55–57]. This suggests that skeletal muscle atrophy is orchestrated by a plethora of signaling molecules in a complicated and multifactorial manner. Therefore, we cannot rule out a possibility that other unknown factors are involved in oleate-mediated prevention of atrophy. Another possible reason explaining this discrepancy is the biphasic nature of ROS. It is well documented that excessive and prolonged sources of oxidant production promote oxidative damage to myofibers, inducing fatigue, insulin resistance, atrophy, and diminished contraction force [58–60]. However, recent accumulating evidences suggested that physiological levels of radicals play an essential role in the maintenance of skeletal muscle, including the control of antioxidant capacity, force production, and myogenesis [40, 61–64]. In our experiment, a mitochondrial-targeted superoxide scavenger, MitoTEMPO, was used to eliminate palmitate-induced mitochondrial ROS. The technical limitation of this method is that it is not possible to scavenge only overproduced portions of superoxide, leading to a possibility that normal and physiological portions of superoxide are also scavenged. This idea is supported by a study proving that MitoTEMPO treatment attenuates the rate of skeletal muscle cell differentiation [40]. On the contrary, a variety of *in vivo* and *in vitro* studies reported positive effects of MitoTEMPO treatment in cardiovascular diseases and insulin resistance in the absence of side effects [65–67]. Thus, our explanation is not yet conclusive, raising a necessity for further study to remove excessive mitochondrial ROS in the absence of neutralizing the ROS necessary for normal physiological function.

**Figure 7:**
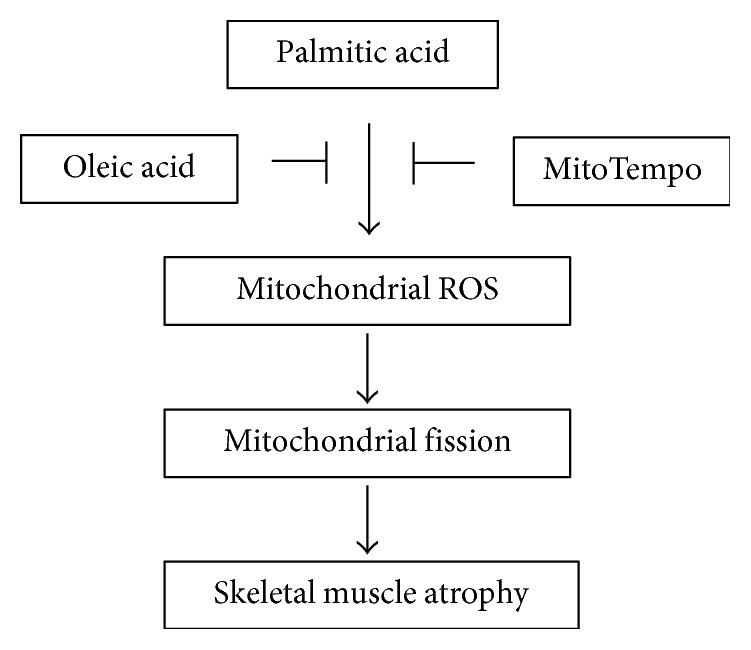
A schematic diagram of the proposed mechanism of oleate-mediated prevention of atrophy in palmitate-rich environment.

Our results indicated that two most abundant fatty acids, palmitate and oleate, affect skeletal muscle integrity in a different way via modulation of the gene expression of mitochondrial fission, proinflammatory cytokines, and atrogenes. Furthermore, our findings also suggested that the fatty acid-mediated mitochondrial redox state is a significant factor in the regulation of myotube atrophy (Figure 7).

## 5. Limitation

Our study demonstrated the protective effect of oleate against palmitate-induced atrophy by incubating the two fatty acids simultaneously. Therefore, with the scope of the current study, it is difficult to rule out a possibility that the effect of oleate might originate from simple uptake competition between oleate and palmitate. However, since palmitate-induced mitochondrial superoxide and dysfunction are completely blocked in neuronal cells by the pre-exposure of oleate alone [30], it is suggested that the effect of oleate in our study was independent of the competition between two fatty acids. And it should be also noted that the myotubes were cultured in the absence of insulin. Accordingly, atrophic characteristics were observed under a relatively low concentration of palmitate (0.3 mM). Considering the fact that insulin has been well documented as a potent regulator of muscle proteolytic pathway [68, 69], myotubes might tolerate a higher dose of palmitate in the presence of insulin. Therefore, a future study is warranted to test the effect of various fatty acids in the presence of insulin under a more physiologically relevant condition.

## 6. Conclusion

The major findings of the study are that oleate prevents the negative effects of palmitate on myotube atrophy and reduced mitochondrial ROS by oleate is at least a significant contributor to the prevention of palmitate-induced atrophy. A greater understanding of *in vivo* mechanisms by which different fatty acids affect skeletal muscle integrity would provide new insights into the development of nutritional and pharmacological strategy for those affected by skeletal muscle-related diseases including sarcopenic obesity and type 2 diabetes.

## Figures and Tables

**Table 1:** Primer sets for qRT-PCR.

Gene		Primer sequence	Gene		Primer sequence
Pgc-1*α*	Forward	TGATGTGAATGACTTGGATACAGACA	Opa1	Forward	GGTCACCACGAGAAATCTCAG
	Reverse	GCTCATTGTTGTACTGGTTGGATATG		Reverse	TCTTCCATTCCGTCTCTAGGTT
Ncor1	Forward	GACCCGAGGGAAGACTACCATT	Drp1	Forward	CGGGACAAGTTAATTCAGGACA
	Reverse	ATCCTTGTCCGAGGCAATTTG		Reverse	GTTCTCGGGCAGACAGTTTTC
Nrf1	Forward	GAACGCCACCGATTTCACTGTC	TNF-*α*	Forward	CACAAGATGCTGGGACAGTGA
	Reverse	CCCTACCACCCACGAATCTGG		Reverse	TCCTTGATGGTGGTGCATGA
Nrf2	Forward	GGCACAGTGCTCCTATGCGTG	IL6	Forward	CCACGGCCTTCCCTACTTC
	Reverse	CCAGCTCGACAATGTTCTCCAGC		Reverse	TTGGGAGTGGTATCCTCTGTGA
Tfam	Forward	CTGATGGGTATGGAGAAGGAGG	IL1-*β*	Forward	GCTCATCTGGGATCCTCTCC
	Reverse	CCAACTTCAGCCATCTGCTCTTC		Reverse	CCTGCCTGAAGCTCTTGTTG
Fis1	Forward	GCTCTAAAGTATGTGCGAGGG	Atrogin-1	Forward	CGTGCCGCCTGGAGAAAC
	Reverse	TGCCTACCAGTCCATCTTTCTT		Reverse	TGGGAGTTGCTGTTGAAGTCG
Mfn1	Forward	TTGATCGAATAGCATCCGAGGA	Murf1	Forward	TGAGGTGCCTACTTGCTCCT
	Reverse	CACAGCATTGCATTGATGACAG		Reverse	TCACCTGGTGGCTATTCTCC
Mfn2	Forward	GTGGGCTGGAGACTCATCG	Myostatin	Forward	TAACCTTCCCAGGACCAGGA
	Reverse	CTCACTGGCGTATTCCGCAA		Reverse	CACTCTCCAGAGCAGTAATT
